# A Retrospective Study of Efficacy and Safety of Albumin-Bound Paclitaxel in Metastatic Breast Cancer

**DOI:** 10.14740/wjon865w

**Published:** 2014-12-03

**Authors:** Rajesh Kumar Singh, Sangeeta Pankaj, Sumit Kumar, Vamshi Rajkota

**Affiliations:** a Regional Cancer Center, Indira Gandhi Institute of Medical Sciences, Patna, India

**Keywords:** Metastatic breast cancer, Retrospective, Nab-paclitaxel, Indian patients

## Abstract

**Background:**

Nab-paclitaxel is a novel nanoparticle, albumin-bound, solvent-free, taxane-based chemotherapy approved for the treatment of metastatic breast cancer (MBC). This study reports clinical benefit and toxicities experienced by women with MBC treated with nab-paclitaxel.

**Methods:**

Women with MBC treated with single-agent nab-paclitaxel between January 2012 and March 2014 were included in this analysis. Retrospective data obtained included demographics, disease characteristics, prior chemotherapy, nab-paclitaxel treatment, toxicity and survival. Clinical benefit was defined as partial or complete response or stable disease (by clinical or radiologic evaluation, or both) at 6 months or more.

**Results:**

Overall response rates (complete or partial responses) were 43% (95% CI: 35.3 - 60.0) for all patients. Median time to disease progression was 26.6 weeks, and median survival was 63.6 weeks. No severe hypersensitivity reactions were reported despite the lack of premedication. Toxicities observed were typical of paclitaxel and included grade 3 sensory neuropathy (14.3%), grade 4 neutropenia (7.14%) and grade 4 febrile neutropenia (7.14%). Patients received a median of six treatment cycles; three patients had 25% dose reductions because of toxicities.

**Conclusions:**

Our clinical experience demonstrates that most women treated with nab-paclitaxel experienced some clinical benefit. Patients achieving clinical benefit lived significantly longer than those who did not. Nab-paclitaxel was well tolerated, with the primary toxicity being mild sensory neuropathy. Nab-paclitaxel represents another treatment option, with a favorable toxicity profile, for women with MBC.

## Introduction

Breast cancer in women is a major health problem worldwide, with approximately 1,676,633 new cases diagnosed and 521,817 deaths attributed to the disease in 2012 [1]. Breast cancer is a heterogeneous disease in terms of gene expression, morphology, clinical course and response to treatment. Gene expression profiling divides breast cancer into several subtypes, including luminal, human epidermal growth factor receptor 2 (HER2)-enriched, basal-like and normal-like subtypes [2]. Luminal A and B subtypes are also classified by expression levels of estrogen receptor (ER)-α and ER-α-related gene. Basal-like subtype is almost identical to triple-negative breast cancer which is defined as showing an absence of ER-α and progesterone receptor, and no protein overexpression or gene amplification of HER2. Luminal A subtype has better prognosis than luminal B, HER2-enriched and basal-like subtypes. Luminal and HER2-enriched subtypes have several effective therapeutic targets such as ER for endocrine therapy and HER2 for anti-HER2 therapy. Basal-like subtype has no effective therapeutic target at present. The response for cytotoxic agents is also different according to tumor subtypes. HER2-enriched and basal-like subtypes are more responsive to chemotherapy than the luminal A subtype [3].

Chemotherapy for patients with breast cancer is effective in not only reducing recurrence of this disease and improving survival in the adjuvant setting, but also in prolonging survival and improving quality of life in the metastatic setting.

Taxanes, such as paclitaxel and docetaxel, are one of the most active and widely used classes of cytotoxic agents in breast cancer treatment. Taxanes act through binding with tubulin and stabilizing non-functional microtubule bundles, leading to subsequent defects in mitotic spindle assembly, chromosome segregation and cell division, resulting in cell death [4, 5]. A meta-analysis showed that the addition of taxane to an anthracycline-based regimen in the adjuvant setting improved the disease-free survival and overall survival (OS) in patients with high-risk early stage breast cancer compared with an anthracycline-based regimen [6]. Another meta-analysis in the metastatic setting showed that taxane and anthracycline were equivalent in terms of effects on response rate and OS, although the taxanes were significantly less efficacious than anthracyclines in relation to impact on progression-free survival (PFS) [7]. However, the conventionally used taxanes, paclitaxel and docetaxel, have several limitations for clinical use. For example, taxanes are poorly soluble in water due to hydrophobic properties, and they require solvents when used in clinical formulations. The use of solvents in the formulation of these therapeutic agents limits their clinical effectiveness, in that these vehicles induce toxic responses, such as hypersensitivity and peripheral neuropathy, and can reduce the extent of drug delivery to tumor [8].

Nanoparticle albumin-bound paclitaxel (nab-paclitaxel, sold under the trade name Abraxane^®^; Celgene Corporation, Summit, NJ), which is a solvent-free form of paclitaxel, can potentially avoid these limitations. Nab-paclitaxel is widely approved for the treatment of metastatic breast cancer (MBC) on the basis of results from pivotal trials showing that it has superior antitumor effects and improved tolerability than solvent based paclitaxel [9]. The clinical efficacy of nab-paclitaxel (260 mg/m^2^ every 3 weeks (q3w)) compared with that of paclitaxel (175 mg/m^2^ q3w) was established in a randomized open-label phase III study of 460 patients with MBC. Most patients in that study had more than three metastatic lesions (76%), a high burden of visceral disease (79%), prior chemotherapy (86%) and progression after first-line therapy (59%). A significant improvement in the overall response rate (33% vs. 19%, P = 0.001) and a longer median time to progression (23.0 weeks vs. 16.9 weeks, P = 0.006) were observed with nab-paclitaxel compared with paclitaxel. The overall study population also showed a trend toward improved median overall survival [9]. Treatment with nab-paclitaxel was well tolerated. Despite a 49% higher paclitaxel dose, the incidence of grade 4 neutropenia was lower with nab-paclitaxel than with solvent-based paclitaxel (9% vs. 22%, P < 0.001), and although the incidence of sensory neuropathy was higher for nab-paclitaxel, that symptom improved rapidly (median: 22 days vs. 79 days) for solvent-based paclitaxel. In this review, we focus on discussing the clinical usefulness, safety and efficacy of nab-paclitaxel. To achieve our aim, we searched papers on nab-paclitaxel for treating breast cancer electronically, by hand and through discussion with experts.

## Materials and Methods

This analysis includes MBC patients treated with single-agent nab-paclitaxel at Regional Cancer Center, Indira Gandhi Institute of Medical Sciences, Patna between January 2012 and March 2014. The data collected included age, date of diagnosis, surgery, estrogen and progesterone receptor status, HER2 status, sites of metastasis, initial tumor stage and lymph node status, histologic diagnosis, endocrine therapy, prior chemotherapy regimens, nab-paclitaxel administration (duration of therapy, dosing schedule, reasons for nab-paclitaxel use), toxicities, dose reductions, clinical benefit and date of death. Toxicities were graded using the Common Terminology Criteria for Adverse Events, version 4.0, based on the description provided in the clinical progress notes.

### Patient population

Non-pregnant women (> 18 years of age) were eligible for inclusion in the study. Patients had histologically or cytologically confirmed breast cancer (measurable bidimensionally) with evidence of recurrence or metastasis but no other malignancy except non-melanoma skin cancer, cervical intraepithelial neoplasia, or *in situ* cervical cancer. Patients had expected survival of > 8 weeks with adequate hematologic, hepatic and renal function. Patients were excluded from participation if they had clinical evidence of brain metastasis; serious concurrent illness; an Eastern Cooperative Oncology Group performance status of ≥ 2; sensory neuropathy grade > 1; or received taxane chemotherapy within the previous 6 months, any other investigational drug within the previous 4 weeks, or anthracycline therapy within the previous 3 weeks. There were no restrictions on enrollment based on the number of prior chemotherapy regimens.

### Therapy

Patients received nab-paclitaxel (260 mg/m^2^) intravenously over 30 min q3w at the discretion of the treating oncologist. Steroid premedication was not required before administration of therapy. Treatment was repeated every 3 weeks until disease progression or unacceptable toxicity occurred. Dose reductions of 25% of the original dose (from 260 to 195 mg/m^2^) were required for grade 4 hematologic toxicity, neutropenic fever or sepsis, or grade 3 or 4 non-hematologic toxicity.

### Clinical benefit

Radiologic and clinical assessments were performed at the discretion of the treating physician. Clinical benefit was measured using the Response Evaluation Criteria in Solid Tumors [10] and was defined as complete response (no clinical or radiologic evidence of disease), partial response (decrease of at least 30% in the sum of lesions) or stable disease (clinically or radiologically, 30% decrease or 20% increase in lesions for a duration of 6 months).

### Statistical analysis

Demographic and clinical data are summarized descriptively as means, medians, or proportions. Survival curves were generated using the Kaplan-Meier method and were compared using the log-rank test. Patients alive or lost to follow-up as of March 31, 2014, were censored. In an exploratory analysis, the relative risk of death for various patient subgroups was estimated using Cox proportional hazards regression. All of the statistical analyses were performed using the Stata software package (release 20.0).

## Results

### Patients

Forty-three patients between January 2012 and March 2014 with MBC received treatment with single-agent nab-paclitaxel at the Regional Cancer Center, IGIMS, Patna and 14 patients were evaluated for clinical response. Table 1 summarizes patient demographics and treatment characteristics. In this group, mean age was 58 years old (range: 39 -70 years), and most patients (71.42%) had estrogen receptor-positive disease. Most patients also had several sites of metastases (71.1%) and had received multiple lines of chemotherapy (median: 3; range: 1 - 6) before receiving nab-paclitaxel.

**Table 1:** Demographic and Treatment Characteristics of Patients With MBC Receiving Nab-Paclitaxel

	Variable value
Patients (n)	14
Age (years)	
Mean	58.0
Range	39 - 70
Receptor status (%)	
ER-positive	71.42
PR-positive	57.14
HER2-positive	14.28
Sites of metastasis (%)	
Bone only	14.28
Liver only	7.14
Lung only	7.14
Multiple sites	71.44
Current line of chemotherapy for advanced disease (median (range))	3 (1 - 6)
Previous taxane exposure (n (%))	10 (71.42)
Adjuvant	2 (20)
Metastatic	7 (70)
Adjuvant and metastatic	1 (10)
Median duration (months)	4.5

ER: estrogen receptor; PR: progesterone receptor; HER2: human epidermal growth factor receptor 2; OS: overall survival.

### Nab-paclitaxel therapy

Nab-paclitaxel treatment was given to 14 patients for a median duration of 4.5 months. Three patients (21.42%) had dose reductions. Reasons for dose reductions included neuropathy (n = 2), decreased body surface area, feeling unwell, decreased Eastern Cooperative Oncology Group performance status and neutropenia (n = 1). Most patients (n = 10, 71.2%) had already been exposed to taxanes in the adjuvant (20%), metastatic (70%), or adjuvant and metastatic (10%) settings.

### Toxicity

Peripheral sensory neuropathy was the most commonly reported toxicity, being reported in approximately 35.7% (n = 5) of all women treated with nab-paclitaxel. Two patients (14.3%) experienced significant (grade 3) peripheral sensory neuropathy. Grade 2 fatigue was reported in 2 patients (14.3%), and grade 3 myalgia, dyspnea, mucositis were each reported in one patient (7.14%). One patient experienced febrile neutropenia.

### Clinical benefit and OS

Clinical benefit was evaluable in 14 patients.

**Figure 1:**
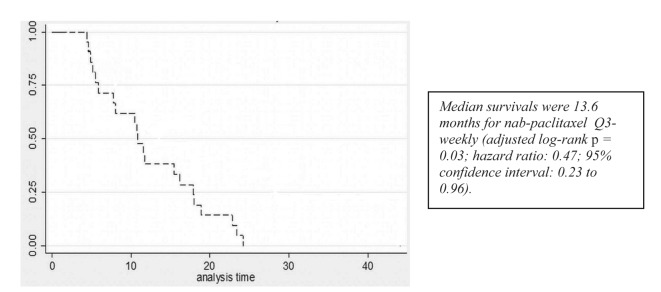
Survival curves for patients receiving q3w nab-paclitaxel.

**Figure 2:**
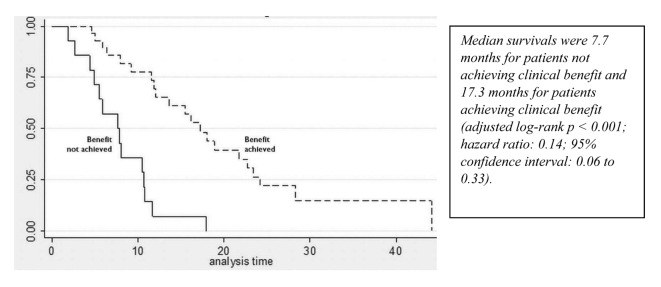
Survival curves for patients who achieved and did not achieve clinical benefit from nab-paclitaxel.

A partial response to nab-paclitaxel therapy was seen in five patients. Women who received nab-paclitaxel q3w had a median OS of 11.9 months in the group (Fig. 1, Table 2). Regardless of the schedule of administration, women who experienced clinical benefit from nab-paclitaxel lived significantly longer than those who did not achieve clinical benefit (17.3 months vs. 7.7 months; hazard ratio (HR): 0.14; 95% CI: 0.06 - 0.33; P < 0.001) (Fig. 2). In the Cox proportional hazards analysis, achievement of clinical benefit (HR: 0.14; P = 0.001) was a predictor of improvement in OS, and exposure to multiple lines of chemotherapy (HR: 1.39; P = 0.021) was associated with a decrease in OS (Table 3). Clinical benefit was achieved in four patients (40.1%) who had prior exposure to taxanes (n = 10). Of the 14 evaluable patients, two (14.2%) progressed while on nab-paclitaxel treatment.

**Table 2:** Clinical Outcomes Data in Patients With MBC Receiving Nab-Paclitaxel (q3w) Schedule

Variable	Nab-paclitaxel schedule q3w (260 mg/m^2^)
Patients (n)	14
Overall response (%)	43
Complete	7.14
Partial	35.71
Development of neuropathy (%)	35.7
Overall survival (months)	
Median	11.9
Interquartile range	7.7 - 21.8

**Table 3:** Results of Cox Proportional Hazards Analysis on OS

Variable	HR	95% CI	P-value	Impact on survival
Line of chemotherapy	1.39	1.05 - 1.84	0.021	↑ Risk by 39% per line
Achievement of clinical benefit	0.14	0.06 - 0.33	0.001	↓ Risk by 86%

## Discussion

Single cytotoxic agents and combination chemotherapy regimens are recommended in NCCN guideline. Preferred single agents includes anthracyclines: doxorubicin, epirubicin; taxanes: paclitaxel, docetaxel and albumin-bound paclitaxel; anti-metabolites: capacitabine and gemcitabine; and non-taxane microtubule inhibitors: erbulin and vinorelbine. Among combination regimen includes: cyclophosphamide, doxorubicin, fluorouracil (FAC/CAF); fluorouracil, epirubicin, cyclophosphamide (FEC); doxorubicin, cyclophosphamide (AC); epirubicin, cyclophosphamide (EC); cyclophosphamide, methotrexate, fluorouracil (CMF); paclitaxel, bevacizumab.

Traditional solvent-based taxanes have been shown to be beneficial in the treatment of MBC. The disadvantage of this group of drugs has been related mainly to the inherent difficulties of drug delivery: the need for solvent, special tubing, and premedication with dexamethasone to prevent allergic reactions. In addition, it has been suggested that the cremophor solvent used in the preparation of paclitaxel may have a direct negative effect on the antitumor properties of that drug [11]. Paclitaxel is entrapped by the formation of plasma cremophor EL micelles, which can cause reduced drug clearance, non-linear pharmacokinetics and free drug fraction [11]. The dose-dependent antitumor response from paclitaxel can be decreased as a result [12]. It is this drug entrapment phenomenon that partly explains why giving higher doses of solvent-based paclitaxel does not result in improved clinical efficacy [13].

Preclinical studies demonstrated that penetration of paclitaxel into tumors was greater with nab-paclitaxel than with solvent based paclitaxel, resulting in superior antitumor activity for nab- paclitaxel in athymic mice with human breast cancer [14, 15]. The use of albumin as the carrier rather than polyethoxylated castor oil allows for doses of paclitaxel much higher than those achievable with paclitaxel, and avoids the toxicities associated with synthetic solvents, as evidenced by the lack of hypersensitivity reactions in the absence of corticosteroid and antihistamine premedication. This albumin-based particle composition of paclitaxel is the first biologically interactive form of the drug with the potential to exploit a receptor-mediated pathway, allowing increased transport of albumin-bound paclitaxel from blood to tumor [16].

Importantly, nab-paclitaxel has demonstrated limited taxane cross-resistance. That feature is of particular clinical importance for women with early stage breast cancer who relapse within 12 months of exposure to a solvent-based taxane. A phase II study[17] of weekly nab-paclitaxel monotherapy in women with taxane-refractory MBC reported 13 partial responses (20%) among 66 evaluable patients, with seven patients responding and three having stable disease (for at least 24 weeks). Those observations suggest that nab-paclitaxel may provide long-term disease control in the difficult-to-treat taxane-refractory MBC population. The issue of lack of cross-resistance is being evaluated more rigorously in the ongoing multicoated single-arm phase II Tiffany trial (search for NCT01416558 at http://clinicaltrials.gov) sponsored by the German Breast Group.

In our small single-institution retrospective study, most women with MBC who received nab-paclitaxel experienced response 42% with the greatest proportion of clinical benefit being observed as stable disease. Notably, clinical benefit was also seen in heavily pretreated women (median of three cycles of chemotherapy before nab-paclitaxel) and those previously exposed to taxanes in the adjuvant and metastatic settings. Women who experienced clinical benefit from nab-paclitaxel survived significantly longer than those who did not (17.3 months vs. 7.7 months, P < 0.001). Nab-paclitaxel was well tolerated, with mild sensory neuropathy (grade 1 or 2) occurring in approximately 27.9% of patients. Dose reductions are mainly because of toxicity. The results of a randomized phase III trial (Cancer and Leukemia Group B (CALGB) 40502/North Central Cancer Treatment Group (NCCTG) N063H) of nab-paclitaxel or ixabepilone or paclitaxel with or without bevacizumab in women with chemotherapy-naive MBC, were recently presented. The second interim analysis demonstrated that nab-paclitaxel was highly unlikely to be superior to paclitaxel for progression-free survival (9.2 months vs. 10.6 months; HR: 1.19; 95% CI: 0.96 - 1.49) [18]. Patients receiving paclitaxel experienced a lower incidence of sensory neuropathy (37% vs. 48%) and fewer hematologic toxicities (12% vs. 49%). Those results differ from the findings reported from the phase III trial by Gradishar et al [9], which demonstrated a superior overall response rate (33% vs. 19%, P = 0.001) and OS (56.4 weeks vs. 46.7 weeks; HR: 0.73; P = 0.024) in the second-line or greater setting with nab-paclitaxel than with paclitaxel. These differences in trial results may reflect different patient populations (chemotherapy-naive vs. pretreated), use of bevacizumab, and varying doses and schedules of taxanes (nab-paclitaxel vs. taxol).

The role of sparc (secreted protein, acidic and rich in cysteine, a form of the caveolin 1 gene) as a biomarker for nab-paclitaxel effectiveness is being explored further in the CALGB 40502/ NCCTG N063H trial (search for NCT00785291 at http://clinicaltrials.gov). That trial will evaluate the relationships of sparc overexpression and of changes in blood levels of caveolin 1 with progression-free survival and secondary end points of response during treatment.

Several limitations in the present study need to be acknowledged. The small study population and retrospective nature of the analysis represent significant limitations in interpreting its results. Clinical benefit was based on clinical and radiologic assessments (for example, computed tomography imaging) of patients, which were requested by the treating physicians at variable points.
